# Left ventricular hypertrophy among adults in a population-based cohort in Haiti

**DOI:** 10.1038/s41598-025-96837-3

**Published:** 2025-04-14

**Authors:** Anju Ogyu, Vanessa Rouzier, Rodney Sufra, Reichling St Sauveur, Marie Christine Jean-Pierre, Joanna Q. Lin, Nour Mourra, Fabiola Preval, Mirline Jean, Richard B. Devereux, Altaf Pirmohamed, Parag Goyal, Lisa de las Fuentes, Victor G. Dávila-Román, Wheytnie Alexandre, Robert N. Peck, Marie-Marcelle Deschamps, Jean W. Pape, Margaret L. McNairy, Lily D. Yan

**Affiliations:** 1 Center for Global Health, Weill Cornell Medicine, New York City, NY USA; 2 Haitian Group for the Study of Kaposi’s Sarcoma and Opportunistic Infections (GHESKIO), Port-au-Prince, Haiti; 3 Division of General Internal Medicine, Weill Cornell Medicine, New York City, NY USA; 4 Division of Cardiology, Weill Cornell Medicine, New York City, NY USA; 5 Global Health Center, Institute for Public Health and Cardiovascular Division, Department of Medicine, Washington University School of Medicine, St. Louis, MO USA; 6 Division of Infectious Disease, Weill Cornell Medicine, New York City, NY USA

**Keywords:** Left ventricular hypertrophy, Cardiovascular, Echocardiography, Electrocardiogram, Cardiovascular diseases, Epidemiology

## Abstract

Left ventricular hypertrophy (LVH) is one of the strongest predictors of cardiovascular disease (CVD) and mortality; yet the means to diagnose LVH in resource-constrained settings remain limited. The objectives of this study were to determine LVH prevalence by transthoracic echocardiography (TTE) in a high-risk group, and compare TTE vs. electrocardiography (ECG-LVH) for LVH detection. We analyzed enrollment data from the Haiti cardiovascular disease cohort study on adults (≥ 18 years, *n* = 3,005) in Port-au-Prince between 2019 and 2021. All participants underwent questionnaires, vital signs, physical exams, and 12-lead ECGs. TTEs were acquired on those with hypertension or exhibiting CVD symptoms (*n* = 1040, 34.7%). TTE-LVH was defined according to the American Society of Echocardiography guidelines and ECG-LVH by Sokolow-Lyon, Cornell, and Limb-Lead Voltage criteria. The prevalence of TTE-LVH was 39.0% (95% CI 36.6–41.5%) and associated with older age. Only 26% of those with TTE-LVH and elevated blood pressure were on antihypertensives. Prevalence of ECG-LVH ranged from 1.9 to 5.0%, and compared to TTE-LVH had low agreement (κ < 0.20), low sensitivity (< 10%) and high specificity (> 90%). These findings indicate a high prevalence of TTE-LVH among high-risk Haitian adults, and poor detection using ECGs compared to TTEs. For those with TTE-LVH, treatment with antihypertensives may reduce the risk of adverse CVD outcomes.

## Introduction

Globally, cardiovascular disease (CVD) is the leading cause of death with over 80% occurring in low-and-middle-income countries (LMIC)^1^. Left ventricular hypertrophy (LVH), an increase in the mass of the left ventricular myocardium, is associated with a two-fold increased risk in both CVD events and all-cause mortality^2^. This risk of LVH is higher in Black adults compared to White adults in studies from the US and other high-income countries^3–6^, with relatively less data available from LMICs^7^. While LVH is typically diagnosed by transthoracic echocardiography (TTEs)^8^, electrocardiography (ECG) is more readily available and affordable in LMIC and has been proposed as a screening tool for LVH .

Haiti is a majority Black nation, the poorest country in the Latin American and Caribbean region, and a country in which CVD is the leading cause of adult death^9^. Our prior studies in Haiti found hypertension, prevalent in 29% of the population, was the leading CVD risk factor with only 13% of Haitians with hypertension having controlled blood pressure. This finding is concerning given that uncontrolled hypertension is a leading risk factor for heart failure, the most common type of CVD previously reported in Haiti^10–12^. LVH is the strongest independent predictor of incident CVD events particularly in people living with hypertension^13^. However, the prevalence of LVH in Haiti is unknown given limited clinical infrastructure for CVD imaging and diagnosis, with only 16 cardiologists serving a population of 11 million^14^. Additionally, the performance of ECG as a diagnostic screen for LVH has not been widely compared to TTEs in a population-based Black cohort located in a LMIC.

To address these gaps, the objectives of this study were to (1) determine LVH prevalence by TTE, and (2) compare TTE vs. electrocardiography for LVH detection (TTE-LVH vs. ECG-LVH) in a high-risk subset of the Haiti Cardiovascular Disease Cohort.

## Methods

### Study design and population

We analyzed baseline data from the Haiti Cardiovascular Disease Cohort, a longitudinal observational study which enrolled 3,005 Black adults between March 2019 and August 2021 aged ≥ 18 years in Port-au-Prince, Haiti at the Groupe Haitien d’Etude Sarcome de Kaposi et des Infections Opportunistes (GHESKIO) clinic. Detailed methodology has been previously described^15^. Multistage random sampling was used to recruit participants from Port-au-Prince. Criteria for inclusion were: age ≥ 18 years, having primary residence in Port-au-Prince, not having serious medical conditions or cognitive impairment and having the ability to speak French or Creole.

**Fig. 1:**
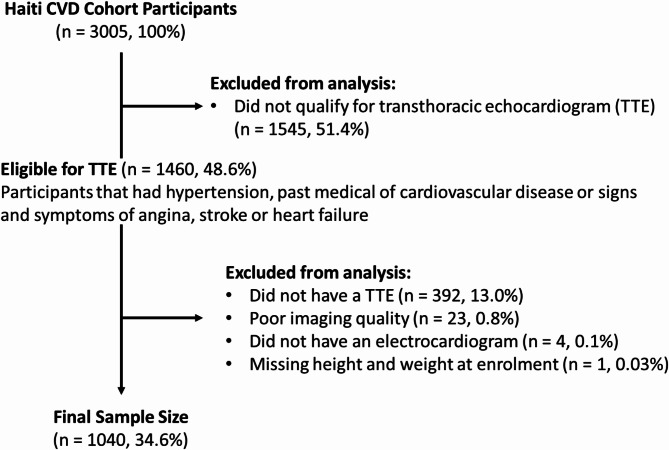
Flow chart of determining the final analytical sample from the Haiti CVD Cohort at enrolment.

This analysis includes a subset of participants who were eligible for screening with both ECG and TTE (Fig. 1). Eligibility for TTE in the parent Haiti cardiovascular disease cohort study was defined as having hypertension, past medical history of CVD, or signs and symptoms of CVD (stroke, angina, or heart failure) met by 1460 (48.6%) participants. A total of 420 (28.8%) participants were excluded because they did not receive TTE (*n* = 392, 13.0%), had poor imaging quality (*n* = 23, 0.8%), missing ECG (*n* = 4, 0.1%), or missing height or weight (*n* = 1, < 0.1%) at baseline (Fig. 1). Characteristics of those who did and did not receive TTE among the 3005 can be found in (Supplementary Table 1).

### Measurements

Sociodemographic data on age, sex, income (< 1 USD/day or ≥ 1 USD/day) and educational attainment (primary or lower, secondary education or higher) were collected. Self-reported health behavior on daily fruit or vegetable intake (< 5 servings/day or ≥ 5 servings/day), smoking status (never/former or current), low alcohol consumption (< 1 drink/day) and moderate weekly physical activity (75 min of vigorous activity or ≥ 150 min of moderate activity per week) were collected using standardized World Health Organization STEPwise approach to NCD risk factor surveillance (WHO STEPS) for comparability^16^.

During a clinical exam, participants were asked about their past medical history (e.g. heart failure, stroke, myocardial infarction, angina, arrhythmia), and current medications in the past two weeks including for diabetes and hypertension. Participants were assessed for CVD signs and symptoms using the Rose Angina Questionnaire, heart failure symptoms based on American Heart Association (AHA) Definitions for Cardiovascular Endpoint Events, and Questionnaire for Verifying Stroke-Free Status (QVSFS)^17–19^.

Vital signs, physical exam, cardiac imaging, and laboratory tests were performed. Blood pressure was collected using OMRON HEM 907 automated oscillometric BP machines (Omron Healthcare, Kyoto, Japan) following guidelines by the AHA and WHO^16,20^. Three readings were collected and the average of the last two readings was used in the analysis. 12-lead ECG was performed using Nasiff CardioCard PC. Participants with hypertension, past medical history of CVD or with signs and symptoms of cardiovascular disease were referred for TTE (Sonosite M-Turbo, P21x/5 − 1 MHz transducer, Fujifilm Sonosite, Inc, Bothell, WA, USA). Laboratory testing included lipids and glucose.

### Cardiovascular disease risk factor definitions

Hypertension was defined using both national and WHO guidelines including systolic blood pressure (SBP) ≥ 140 mmHg or diastolic blood pressure (DBP) ≥ 90 mmHg or taking antihypertensives, pre-hypertension as 120–139 mmHg or DBP 80–89 mmHg, and normal BP as SBP < 120 mmHg and DBP < 80 mmHg^21^. Body mass index (BMI) was calculated using weight (kg)/height (m)^2^ and categorized as underweight (BMI < 18.5), normal (BMI 18.5–24.9), overweight (BMI 25.0-29.9) and obese (BMI ≥ 30). Diabetes mellitus was defined as fasting glucose ≥ 126 mg/dL, non-fasting glucose ≥ 200 mg/dL, or taking antidiabetic medications. Hypercholesterolemia was defined as either total cholesterol ≥ 240 mg/dL, low-density lipoprotein level ≥ 160 mg/dL or taking statins^22^. Arrhythmia was abstracted from physician diagnosis codes.

### Echocardiography

For TTE-LVH, linear left ventricular dimensions and wall thickness (septum and posterior wall) were acquired from two-dimensional TTE images in the parasternal long axis view at end-diastole. LV mass was calculated using the linear method Devereux formula which was selected due to its ease of acquisition relative to other two-dimensional volumetric methods, concordance with autopsy LV mass, and its common use in other cohort studies thus allowing comparisons across populations^23,24^. The Mosteller formula for body surface area (BSA) was calculated using the participant’s height and weight at the time of imaging (½ (height (cm) x weight (kg)/3600))^25^. LV mass was indexed to BSA (LVMI). TTE-LVH was defined as an LVMI > 95 g/m^2^ for females and > 115 g/m^2^ for males based on American Society of Echocardiography (ASE) 2015 Chamber Quantification Guidelines^24^. Relative wall thickness (RWT) was calculated as (2 x LV posterior wall thickness at end-diastole)/LV internal dimension at end-diastoled with value ≤ 0.42 defined as normal. Patterns of geometric changes in the left ventricle were classified as normal (normal LVMI and normal RWT), concentric remodeling (normal LVMI and increased RWT), concentric hypertrophy (increased LVMI and increased RWT) and eccentric hypertrophy (increased LVMI and normal RWT)^24^.

ECG-LVH was defined using established criteria: Sokolow-Lyon and Cornell voltage criteria were selected as they are commonly used clinically and often reported in the literature;^26^ and Limb-lead voltage, a single-lead criterion, was used to determine the relative performance of this simpler assessment. Sokolow-Lyon criteria defines LVH as sum of the S peak in V_1_ and R peak in V_5/6_ >38 mm, Cornell voltage criteria defines LVH as the sum of the S peak in V_3_ and the R peak in aVL ≥ 28 mm in men and ≥ 20 mm in women, and Limb Lead criteria defines LVH as the R peak in aVL ≥ 11 mm^16,17^. We also investigated the additive effect of combining different ECG criteria where LVH was defined as having met the LVH definition for at least one of the three ECG criteria.

### Statistical analysis

Descriptive statistics were calculated including mean (standard deviation), counts and percentages. Prevalence of LVH was determined separately for TTE, individually for each of the three ECG criteria, and by a composite ECG variable (i.e., meeting at least one of the three ECG criteria). Prevalent risk factors were compared between LVH vs. no LVH based on TTE definition using Wilcoxon rank sum test for continuous variables, chi-squared or Fisher’s exact test for categorical variables as appropriate. We performed multivariable Poisson regression with robust standard errors to calculate prevalence ratios for TTE-LVH, adjusting for age, sex, education level, income level, physical activity and BMI.

To compare the diagnostic performance of ECG versus TTE in detection of LVH, we calculated the Cohen’s Kappa statistic to measure agreement between each ECG criteria (individual and composite) and TTE as the primary analysis^27^. In addition, we evaluated the sensitivity and specificity of each ECG criteria against TTE, our reference standard^28^. All statistical analyses were conducted using R Version 4.3.2.

### Ethics

This study was approved by the Weill Cornell Medicine Institutional Review Board (record number 1803019037), and the Groupe Haitien d’Etude du Sarcome de Kaposi et des Infections Opportunistes (GHESKIO) Comité des Droits Humains (record number 1803019037). Written informed consent was obtained from all subjects. All research was performed in accordance with the Declaration of Helsinki.

## Results

At enrollment, 48.6% (*n* = 1,460) of the 3,005 participants qualified for TTE, of whom 1089 (36.2%) underwent echocardiography, and 1,040 (34.6%) had data available for analysis (Fig. 1). In comparing participants who did and did not receive TTE within the entire cohort, participants who underwent TTE were more likely to be female and to have lower education attainment (Supplementary Table 1). Among those who did receive TTE, a majority were female (67.1%) with a mean age of 48.5 years (SD = 15.3). More than half (62.6%) were hypertensive of which 33.1% reported taking antihypertensives (Table 1). Among non-hypertensive individuals with TTE, 47.0% (*n* = 183/389) reported with signs and symptoms of angina, heart failure, myocardial infarction, or stroke and the remaining participants were selected for TTE based on other clinical criteria.

**Table 1:** Characteristics of participants without and with LVH based on transthoracic echocardiography.

Characteristic	Overall *N* = 1040 (100.0%)	No LVH *N* = 634 (60.9%)	With LVH *N* = 406 (39.0%)	*p*-value^2^
Demographics				
Sex, female	698 (67.1)	422 (66.6)	276 (67.98)	0.6
Age (mean, SD)	48.5 (15.3)	45.6 (15.6)	53.0 (13.8)	< 0.001
Age, years				< 0.001
18–29	159 (15.3)	133 (21.0)	26 (6.4)	
30–39	147 (14.1)	100 (15.8)	47 (11.6)	
40–49	210 (20.2)	134 (21.1)	76 (18.7)	
50–59	235 (22.6)	127 (20.0)	108 (26.6)	
60+	289 (27.8)	140 (22.1)	149 (36.7)	
Daily income, < 1 USD/day	748 (71.9)	460 (72.6)	288 (70.9)	0.6
Education				< 0.001
< Secondary	524 (50.5)	291 (46.0)	233 (57.5)	
≥ Secondary	513 (49.5)	341 (54.0)	172 (42.5)	
Unknown	3	2	1	
Health behaviours				
Fruit and vegetable intake				0.2
< 5 servings a day	1033 (99.6)	628 (99.4)	405 (100.0)	
≥ 5 servings a day	4 (0.4)	4 (0.6)	0 (0.0)	
Unknown	3	2	1	
Smoking status				0.8
Current	43 (4.2)	27 (4.3)	16 (4.0)	
Never/former	987 (95.8)	602 (95.7)	385 (96.0)	
Unknown	10	5	5	
Alcohol intake				0.6
< 1 drink a day (low)	1005 (97.1)	614 (97.3)	391 (96.8)	
≥ 1 + drink a day (moderate or higher)	30 (2.9)	17 (2.7)	13 (3.2)	
Unknown	5	3	2	
Physical activity				0.4
≤ 150 min/week (low)	496 (47.9)	309 (49.0)	187 (46.3)	
> 150 min/week (moderate-high)	539 (52.1)	322 (51.0)	217 (53.7)	
Unknown	5	3	2	
CVD risk factors				
SBP (mean, SD)	138.6 (26.6)	132.2 (24.6)	148.6 (26.6)	< 0.001
DBP (mean, SD)	82.8 (16.7)	79.5 (15.9)	88.0 (16.6)	< 0.001
Blood pressure classification^3^				< 0.001
Normal	250 (24.0)	200 (31.6)	50 (12.3)	
Pre-hypertension	139 (13.37)	105 (16.6)	34 (8.4)	
Hypertension	651 (62.6)	329 (51.9)	322 (79.3)	
Taking antihypertensives	216 (20.8)	110 (17.4)	106 (26.1)	< 0.001
BMI category				0.065
Underweight	47 (4.5)	33 (5.2)	14 (3.5)	
Normal	433 (41.6)	279 (44.0)	154 (37.9)	
Overweight	318 (30.6)	187 (29.5)	131 (32.3)	
Obese	242 (23.3)	135 (21.3)	107 (26.4)	
Diabetes mellitus	92 (8.9)	48 (7.6)	44 (10.8)	0.070
Hypercholesterolemia	184 (17.7)	102 (16.1)	82 (20.2)	0.090

^1^n (%); mean (SD).

^2^Pearson’s Chi-squared test; Wilcoxon rank sum test.

^3^Blood pressure was classified as: Normal (SBP < 120 and DBP < 80), pre-hypertension (SBP 120 to 139 or DBP 80 to 89) and hypertensive (SBP ≥ 140 or DBP ≥ 90 or taking antihypertensive).

### LVH prevalence and risk factors

**Fig. 2:**
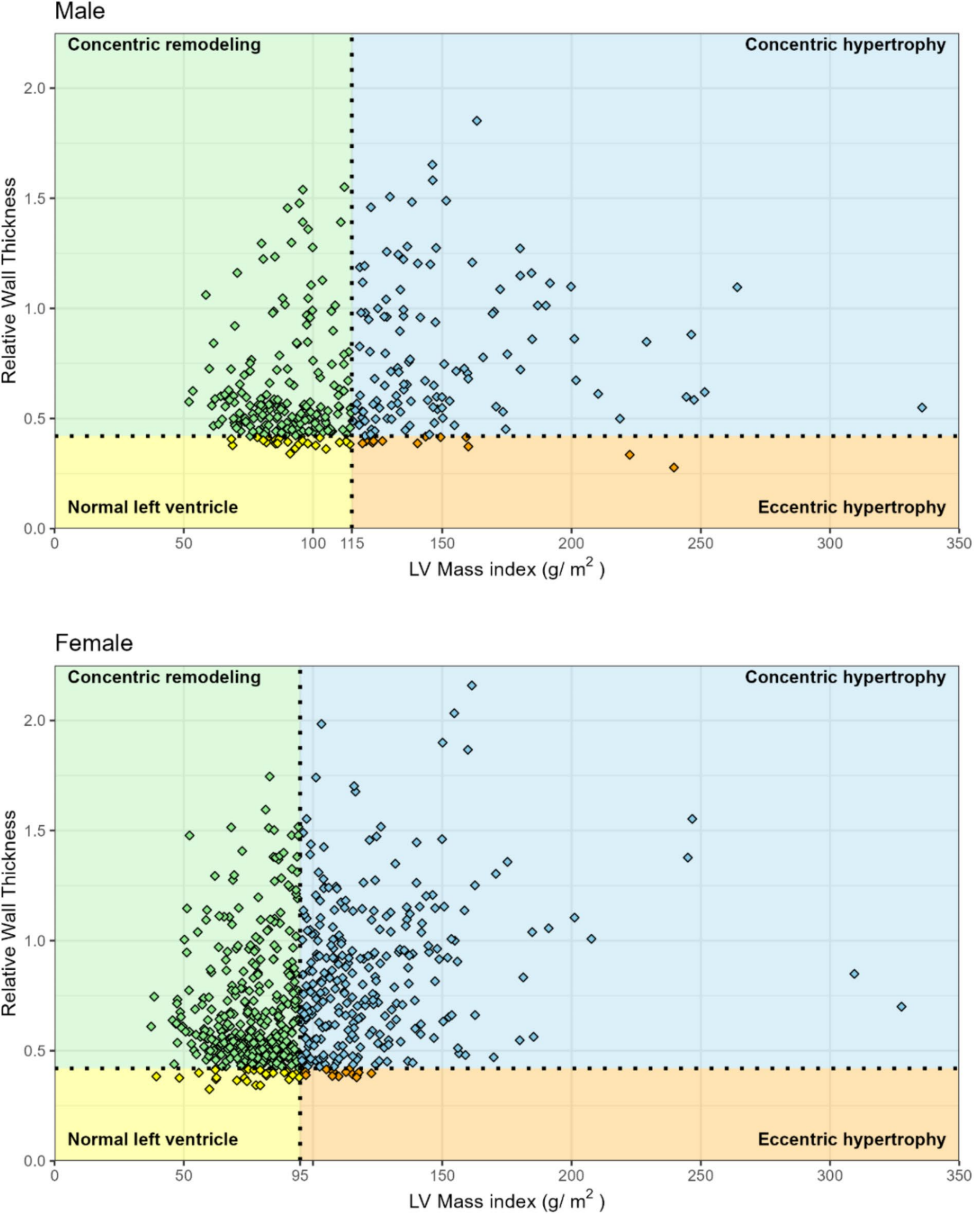
Left ventricular mass index (LVMI) plotted against relative wall thickness (RWT) in all participants with echocardiography. The vertical dotted line represents the cut-off values for normal LVMI (< 95 g/m^2^ in women and < 115 g/m^2^ in men) and the horizontal dashed line for RWT (normal < 0.42).

The prevalence of LVH as defined by TTE was 39.0% (95% CI 36.6, 41.5) overall with 39.5% (95% CI 36.5, 42.6) in females and 38.0% (95% CI 33.7, 42.3) in males. In univariable analysis, participants with LVH were more likely to be older (mean age 45.6 vs. 53.0 years), to only have primary education, have higher mean SBP and DBP, have hypertension, take antihypertensive medication or had been diagnosed with an arrythmia. LVMI among those with LVH ranged from 95.3 to 328.0 g/m^2^ (median, IQR = 88.6, 33.5) in females and 115.0–336.0 g/m^2^ (median, IQR = 103.0, 44.4) in males (Fig. 2). In multivariable regression, there was a stepwise increase in LVH prevalence with older age: participants 60 + years vs. 18–29 years had an adjusted LVH prevalence ratio of 3.26 (95% CI 2.18, 4.88) (Table 2). Normal left ventricle geometry was found in 52 (5.0%), whereas 582 (56.0%) had concentric remodeling. Among those with increased LVMI, 380 (93.6%) had concentric hypertrophy and 26 (2.5%) had eccentric hypertrophy (Supplementary Table 2).

**Table 2:** Multivariable Poisson regression to calculate the adjusted prevalence ratio of TTE-LVH in a high-risk group of Haitian adults using robust standard errors.

Characteristics	Adjusted prevalence ratio (95% CI)	*p*-value
Sex, male	0.97 (0.81, 1.16)	0.712
Age		
18–29	*Ref.*	
30–39	1.87 (1.22, 2.87)	< 0.01
40–49	2.09 (1.39, 3.14)	< 0.001
50–59	2.75 (1.84, 4.11)	< 0.001
60+	3.26 (2.18, 4.88)	< 0.001
Income (≥ 1 USD/day)	1.03 (0.88, 1.22)	0.705
Education level (≥ secondary)	1.07 (0.9, 1.28)	0.425
Physical activity level (> 150 min/week)	1.04 (0.89, 1.21)	0.632
BMI category		
Underweight/normal	*Ref.*	
Overweight	1.11 (0.92, 1.33)	0.272
Obese	1.18 (0.96, 1.45)	0.107

### ECG vs. TTE detection of LVH

**Fig. 3:**
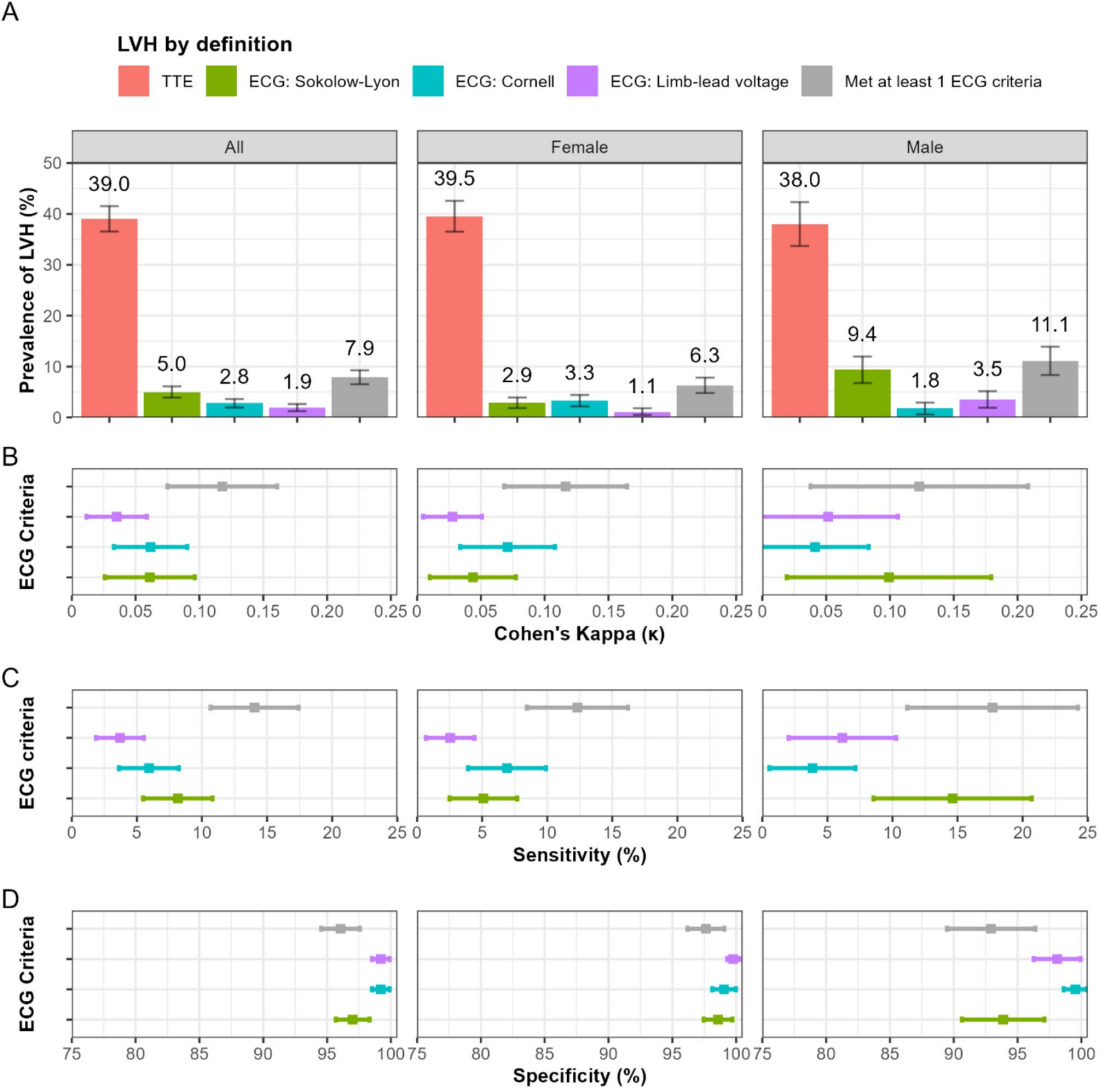
(**A**) Prevalence of left ventricular hypertrophy (LVH) based on the five diagnostic methods. (**B**) Cohen’s kappa statistic, (**C**) sensitivity and (**D**) specificity of each ECG criteria compared with reference standard transthoracic echocardiography for diagnosing LVH. Error bars represent the 95% confidence interval.

The prevalence of LVH by single ECG criterium in both sexes based on the Sokolow-Lyon, Cornell voltage criteria and Limb-lead criteria was 5.0, 2.8 and 1.9%, respectively. In females, the prevalence ranged from 1.1 to 5.0% and for males 1.8–9.4% (Fig. 3A). Prevalence of LVH by meeting at least one of the three criteria compared to using single criteria was significantly higher overall (7.9%) and in females (6.3%) but did not differ in males for Sokolow-Lyon.

Agreement on LVH categorization between TTE and Sokolow-Lyon criteria was κ = 0.06 (95%CI: 0.03, 0.10), Cornell voltage criteria was κ = 0.06 (95% CI 0.04, 0.09), Limb-lead was κ = 0.04 (95%CI: 0.01, 0.06) and for the composite ECG criteria was κ = 0.12 (95% CI 0.08, 0.16) (Fig. 3B). Compared to the reference standard measurement of TTE, the sensitivity of Sokolow-Lyon criteria was 8.2% (95% CI 5.5, 10.8), Cornell voltage criteria was 5.9% (95%CI: 3.6, 8.2) and Limb-lead was 3.7% (95% CI 1.9, 5.5) (Fig. 3C). LVH detection by meeting at least one of the three ECG criteria only modestly improved sensitivity to 14.0% (10.7, 17.4). In contrast the specificity of Sokolow-Lyon criteria was 97.0% (95% CI 95.7, 98.3), Cornell voltage criteria was 99.2% (95% CI 98.5, 99.9) and Limb-lead was 99.2% (95% CI 98.5, 99.9) (Fig. 3D); however, the composite ECG criteria had reduced specificity to 96.1% (95% CI 94.5, 97.6). In females, sensitivity and specificity of the three different ECG criteria ranged from sensitivity of 2.9−7.7% and specificity of 98.4−99.7%. In males, the Cornell voltage criteria and Limb Lead performed similarly in both sensitivity and specificity (Fig. 3).

## Discussion

Among a cohort of Haitian Black adults with hypertension or reporting cardiac symptoms, the prevalence of echocardiography-determined LVH was high at 39.0%. In contrast, LVH defined by three different ECG criteria ranged from 1.9 to 5.9%. All ECG criteria had low agreement with TTE (κ < 0.20), low sensitivity < 15% compared to TTE, and high specificity > 90%. These findings suggest that ECG could be used to detect some cases of LVH but TTEs may be needed to systematically identify LVH even among individuals with hypertension and/or cardiac symptoms.

Our data provides previously unknown estimates of LVH among a high-risk subset of a population based cohort of Black Haitians. Our results are similar to epidemiological findings on hypertensive patients in clinic-based cohorts in Jamaica, Tanzania, Nigeria, Cameroon and the Republic of Seychelles where prevalence of LVH ranged from 15–62%.^29–34^ However, these participants from other studies were older (49 vs. 56 years) or were a convenience sample of patients from cardiology clinics seeking treatment. In studies examining Black normotensive participants based in Angola, Mauritius and the Republic of Seychelles, prevalence of LVH using either ECG or TTE ranged from 6–41%.^32,35,36^

Our findings of low agreement, low sensitivity and high specificity of ECG-detected LVH compared to TTE are consistent with other studies that report similar findings in Black populations living in Europe and the US^37–43^. Our study shows improvement in the sensitivity of LVH diagnosis by ECG by combining the usage of these three criteria, however agreement with TTE-LVH remained low. In addition, our three screening criteria for TTE was able to detect LVH in 40% of the participants suggesting that the combination of BP reading, screening questions for CVD symptoms and visually assessing for abnormal ECGs are helpful in identifying and prioritizing patients for TTE^44,45^.

It is well established that LVH predicts CVD morbidity, cardiac death, and all-cause mortality independent of traditional CVD risk factors in both asymptomatic and symptomatic individuals^46–50^. LVH is associated with a 4-fold increased risk of fatal-stroke^51^, 40–80% increased risk of heart failure^52^ and also has been linked to a 2-fold increased risk of dementia^53^. Findings from the MAVI study conducted in an all-White cohort in Italy with 3-years of median follow-up showed a strong continuous relationship with a 40% increased risk of CVD events for every 39 g/m^2^ increase in LVMI^46^. Our data reports LVH among a cohort of high risk Black Haitians living in extreme poverty and an important next step will be to assess LVH with incident cardiac events to determine if the relationship between LVH and poor CVD outcomes is similar in settings of extreme poverty as the “floor” of the global poverty continuum.

While the gold standard diagnostic study for LVH is cardiac magnetic resonance imaging, ECG is far more affordable, widely available, and require less skill to obtain in primary care settings^23,54^. However, interpretation and accuracy of results is variable. Currently, 36 criteria for LVH have been endorsed by the AHA with no single criterion recommended to guide clinical care^55^. Methods to improve detection of LVH using ECGs through machine learning algorithms conducted across multiple studies located in North America, East Asia and Europe have improved sensitivity but failed to outperform specificity of existing ECG criteria^56^. Similar results were found when adjusting ECG criteria with BMI or BSA improved sensitivity by 2-fold but at the expense of reduction in specificity^33^. A new proposed method by Peguero et al. involving the measurement of the deepest S wave and S wave amplitude of lead V_4_ showed significantly higher sensitivity compared to Cornell voltage (62 vs. 35%) whilst maintaining high specificity of ≥ 90%^57^. A meta-analysis of six studies utilizing this new criteria showed a pooled sensitivity of 56% and specificity of 90%^58^.

Detection of LVH in low-income settings may be important to identify high risk individuals to target for prevention of CVD-related morbidity and mortality. Treatment of patients with LVH should focus on the underlying cause, including treating hypertension to achieve BP control, or valve replacement for aortic stenosis^59^. However, achieving these outcomes in Haiti and other LMICs is difficult with multilevel barriers at the individual, community, and societal levels^60–62^. In Haiti, we have previously shown only 45% of people with hypertension are receiving treatment and only 13% achieve BP control^12^. In this analysis, among those with TTE-LVH, while 87% had elevated blood pressure, only 26% were on antihypertensive medications, pointing to a gap in treatment.

Strengths of our study include being the first study examining the epidemiology of LVH of Haitians living in Port-au-Prince using both TTE and three commonly used ECG criteria. We further classified the cohort by left ventricle geometry. We identified individuals who were normotensive or pre-hypertensive who nonetheless met criteria for TTE-LVH. In these individuals, processes other than hypertension, such as cardiomyopathy or rheumatic heart disease, may be contributing to increased LVM.

The design of this study presents some limitations. First, we were unable to estimate the population prevalence of LVH given TTE was only obtained in a high-risk subset of participants with hypertension or CVD. Second, differences in social and environmental contexts in Haiti versus other world populations may limit comparisons between groups.

In conclusion, more than a third of individuals with hypertension or reporting CVD symptoms in a population-based cohort in Haiti had LVH as detected using TTE. Furthermore, LVH detection using ECG had poor agreement with detection using TTE, as well as low sensitivity and high specificity, with minimal improvements in all measures from the additive effect of composite ECG criteria. These results suggest the diagnosis of LVH likely still requires TTE in low-resource settings. Increasing TTE capacity with training and education should be prioritized for LMICs where burden of CVD is growing fastest. Further research is needed to better understand whether interventions specifically targeting Black individuals with LVH result in improved CVD outcomes.

## Electronic supplementary material

Below is the link to the electronic supplementary material.

Supplementary Material 1

## Data Availability

Researchers who provide a methodologically sound proposal may have access to a subset of deidentified participant data, with specific variables based on the proposal. Proposals should be directed to the principal investigator at mam9365@med.cornell.edu. To gain access, data requestors will need to sign a data access agreement. Data are available following publications through 3 years after publication and will be provided directly from the PI.
