# Supplementary material for: Left ventricular hypertrophy among adults in a population-based cohort in Haiti
Source: Sci Rep. 2025 Apr 14;15:12831. doi: 10.1038/s41598-025-96837-3 (PMC11997160; doi:10.1038/s41598-025-96837-3)
Supplement: Supplementary file 1 — Supplementary Material 1 [file 41598_2025_96837_MOESM1_ESM.docx]

| **Supplementary Table 1 – Characteristics of participants who received an echocardiogram at baseline in the Haiti CVD Cohort.** | | | | |
| --- | --- | --- | --- | --- |
| **Characteristic** | **Overall** N = 3,005*^1^* | **Did not get echocardiogram** N = 1,916*^1^* | **Had echocardiogram** N = 1,089*^1^* | **p-value***^2^* |
| **Demographics** |  |  |  |  |
| **Sex, female** | 1,745 (58.1) | 1,019 (53.2) | 726 (66.7) | <0.001 |
| **Age (Mean, SD)** | 41.8 (15.9) | 38.1 (14.9) | 48.4 (15.5) | <0.001 |
| **Age, years** |  |  |  | <0.001 |
| 18-29 | 890 (29.6) | 720 (37.6) | 170 (15.6) |  |
| 30-39 | 569 (18.9) | 416 (21.7) | 153 (14.1) |  |
| 40-49 | 533 (17.7) | 312 (16.3) | 221 (20.3) |  |
| 50-59 | 499 (16.6) | 258 (13.5) | 241 (22.1) |  |
| 60+ | 514 (17.1) | 210 (11.0) | 304 (27.9) |  |
| **Daily Income, <1 USD/day** | 2,114 (70.4) | 1,330 (69.4) | 784 (72.0) | 0.14 |
| **Education** |  |  |  | <0.001 |
| Primary or lower | 1,073 (35.8) | 532 (27.8) | 541 (49.9) |  |
| Secondary or higher | 1,923 (64.2) | 1,379 (72.2) | 544 (50.1) |  |
| Unknown | 9 | 5 | 4 |  |
| **Health Behaviour** |  |  |  |  |
| **Fruit and vegetable intake** |  |  |  | 0.3 |
| < 5 servings a day | 2,974 (99.3) | 1,894 (99.2) | 1,080 (99.5) |  |
| ≥ 5 servings a day | 20 (0.7) | 15 (0.8) | 5 (0.5) |  |
| Unknown | 11 | 7 | 4 |  |
| **Smoking status** |  |  |  | 0.3 |
| Current | 107 (3.6) | 63 (3.3) | 44 (4.1) |  |
| Never/Former | 2,874 (96.4) | 1,840 (96.7) | 1,034 (95.9) |  |
| Unknown | 24 | 13 | 11 |  |
| **Alcohol intake** |  |  |  | 0.055 |
| < 1 drink a day (low) | 2,876 (96.3) | 1,824 (95.8) | 1,052 (97.1) |  |
| ≥ 1+ drink a day (moderate or higher) | 112 (3.8) | 81 (4.3) | 31 (2.9) |  |
| Unknown | 17 | 11 | 6 |  |
| **Physical activity** |  |  |  | 0.3 |
| ≤ 150 min/week (low) | 1,517 (50.7) | 955 (50.1) | 562 (51.9) |  |
| > 150 min/week (moderate-high) | 1,474 (49.3) | 953 (50.0) | 521 (48.1) |  |
| Unknown | 14 | 8 | 6 |  |
| **CVD Risk Factors** |  |  |  |  |
| **SBP (Mean, SD)** | 125.6 (24.2) | 118.5 (19.4) | 138.1 (26.6) | <0.001 |
| **DBP (Mean, SD)** | 75.0 (15.5) | 70.8 (13.0) | 82.5 (16.6) | <0.001 |
| Unknown | 2 | 2 | 0 |  |
| **Blood Pressure Classification^3^** |  |  |  | <0.001 |
| Normal | 1,388 (46.2) | 1,120 (58.5) | 268 (24.6) |  |
| Pre-hypertension | 636 (21.2) | 486 (25.4) | 150 (13.8) |  |
| Hypertension | 979 (32.6) | 308 (16.1) | 671 (61.6) |  |
| Unknown | 2 | 2 | 0 |  |
| **Taking antihypertensives** | 306 (10.2) | 81 (4.2) | 225 (20.7) | <0.001 |
| **BMI Category** |  |  |  | <0.001 |
| Underweight | 144 (4.8) | 95 (5.0) | 49 (4.5) |  |
| Normal | 1,557 (51.9) | 1,103 (57.7) | 454 (41.7) |  |
| Overweight | 784 (26.1) | 451 (23.6) | 333 (30.6) |  |
| Obese | 515 (17.1) | 263 (13.8) | 252 (23.2) |  |
| Unknown | 5 | 4 | 1 |  |
| **Arrhythmia** | 36 (1.2) | 8 (0.4) | 28 (2.6) | <0.001 |
| **Diabetes Mellitus** | 159 (5.3) | 65 (3.4) | 94 (8.6) | <0.001 |
| Unknown | 5 | 5 | 0 |  |
| **Hypercholesterolemia** | 371 (12.4) | 180 (9.4) | 191 (17.5) | <0.001 |
| Unknown | 7 | 7 | 0 |  |
| **CVD Symptoms** | 816 (27.2) | 356 (18.6) | 460 (42.2) | <0.001 |
| Stroke | 61 (2.0) | 22 (1.2) | 39 (3.6) | <0.001 |
| Heart Failure | 597 (19.9) | 238 (12.4) | 359 (33.0) | <0.001 |
| Myocardial Infarction | 15 (0.5) | 8 (0.4) | 7 (0.6) | 0.4 |
| Angina | 304 (10.1) | 139 (7.3) | 165 (15.2) | <0.001 |
| **Risk** |  |  |  | <0.001 |
| Low-Risk | 1545 (51.4) | 1524 (79.5) | 21 (1.9) |  |
| High-Risk | 1460 (48.6) | 392 (20.5) | 1068 (98.1) |  |
| **ECG-LVH by criteria** |  |  |  |  |
| Any of the three | 152 (5.1) | 69 (3.6) | 83 (7.6) | <0.001 |
| Sokolow-Lyon | 107 (3.6) | 55 (2.9) | 52 (4.8) | 0.008 |
| Missing | 35 | 29 | 6 |  |
| Cornell | 37 (1.3) | 8 (0.4) | 29 (2.7) | <0.001 |
| Missing | 36 | 30 | 6 |  |
| Limb-lead | 32 (1.1) | 11 (0.6) | 21 (1.9) | <0.001 |
| Missing | 41 | 34 | 7 |  |
| *^1^*n (%); Mean (SD) | | | | |
| *^2^*Pearson's Chi-squared test; Wilcoxon rank sum test  ^3^ Blood pressure was classified as: Normal (SBP < 120 and DBP < 80), pre-hypertension (SBP 120 to 139 or DBP 80 to 89) and hypertensive (SBP ≥ 140 or DBP ≥ 90 or taking antihypertensive) | | | | |

|  | **Overall**  N = 1040 (100%) | **Normal left ventricle**  N = 52 (5.0%) | **Concentric Remodeling**  N = 582 (56.0%) | **Concentric Hypertrophy**  N = 380 (36.5%) | **Eccentric Hypertrophy**  N = 26 (2.5%) | **p-value** |
| --- | --- | --- | --- | --- | --- | --- |
| **Age (years)** | 48.5 (15.3) | 40.4 (14.4) | 46.0 (15.6) | 53.2 (13.8) | 50.0 (13.2) | <0.001 |
| **Sex, Female** | 698 (67.1) | 29 (55.8) | 393 (67.5) | 263 (69.2) | 13 (50.0) | 0.063 |
| **Body Surface Area (m^2^)** | 1.7 (0.2) | 1.8 (0.2) | 1.7 (0.2) | 1.8 (0.2) | 1.8 (0.2) | 0.003 |
| **Body Mass Index (kg/m^2^)** | 26.3 (5.8) | 26.9 (6.8) | 25.8 (5.9) | 26.9 (5.6) | 26.6 (5.0) | 0.018 |
| **Systolic BP (mmHg)** | 138.6 (26.6) | 125.8 (22.9) | 132.8 (24.7) | 149.0 (26.7) | 142.9 (24.3) | <0.001 |
| **Diastolic BP (mmHg)** | 82.8 (16.7) | 74.9 (17.4) | 79.9 (15.7) | 88.0 (16.6) | 87.7 (16.7) | <0.001 |
| **Posterior Wall Thickness (cm)** | 1.3 (0.4) | 0.9 (0.1) | 1.2 (0.3) | 1.5 (0.4) | 1.0 (0.1) | <0.001 |
| **Diastolic Dimension (cm)** | 3.8 (0.7) | 4.7 (0.4) | 3.7 (0.6) | 3.8 (0.7) | 5.1 (0.5) | <0.001 |
| **Relative Wall Thickness (cm)** | 0.7 (0.3) | 0.4 (0.02) | 0.7 (0.26) | 0.9 (0.33) | 0.4 (0.03) | <0.001 |
| **Left Ventricular Mass Index (g/m^2^)** | 100 (35) | 83 (15) | 80 (14) | 131 (35) | 130 (34) | <0.001 |
| **Left Ventricular Mass (g)** | 175 (65) | 151 (33) | 138 (29) | 230 (67) | 232 (57) | <0.001 |
| **Stroke Index (ml/m^2^)** | 35 (15) | 38 (14) | 32 (13) | 36 (15) | 52 (19) | <0.001 |
| Missing | 9 | 1 | 4 | 3 | 1 |  |
| **Cardiac Index (l/min per m^2^)** | 2.5 (1.2) | 2.6 (1.1) | 2.3 (1.0) | 2.7 (1.2) | 3.8 (1.5) | <0.001 |
| Missing | 72 | 6 | 39 | 25 | 2 |  |
| **Ejection Fraction (%)** | 64 (9) | 66 (7) | 64 (8) | 63 (11) | 60 (11) | 0.072 |
| **EF <52%** | 284 (27.3) | 9 (17.3) | 145 (24.9) | 123 (32.5) | 7 (26.9) | 0.025 |
| Missing | 1 | 0 | 0 | 1 | 0 |  |

**Supplementary Table 2 – Characteristics of participants based on left ventricular geometry classification**
